# Expression of Glutamine Metabolism-Related and Amino Acid Transporter Proteins in Adrenal Cortical Neoplasms and Pheochromocytomas

**DOI:** 10.1155/2021/8850990

**Published:** 2021-01-05

**Authors:** Hye Min Kim, Ja Seung Koo

**Affiliations:** Department of Pathology, Yonsei University College of Medicine, Seoul, Republic of Korea

## Abstract

**Background:**

Glutamine metabolism is considered an important metabolic phenotype of proliferating tumor cells.

**Objective:**

The objective of this study was to investigate the expression of glutamine metabolism-related and amino acid transporter proteins in adrenal cortical neoplasms (ACNs) and pheochromocytomas (PCCs) in the adrenal gland.

**Methods:**

A tissue microarray was constructed for 132 cases of ACN (115 cases of adrenal cortical adenoma and 17 cases of adrenal cortical carcinoma) and 189 cases of PCC. Immunohistochemical staining for glutamine metabolism-related proteins GLS1 and GDH and amino acid transporter proteins SLC1A5, SLC7A5, and SLC7A11 as well as SDHB was performed and compared with clinicopathologic parameters.

**Results:**

The expression levels of GLS (*p* < 0.001), SLC7A5 (*p* = 0.049), and SDHB (*p* = 0.007) were higher in ACN than in PCC, whereas the expression levels of SLC1A5 (*p* < 0.001) and SLC7A11 (*p* < 0.001) were higher in PCC than in ACN. In ACN, GLS positivity was associated with a higher Fuhrman grade (*p* = 0.009), and SLC1A5 positivity was associated with SDHB positivity (*p* = 0.004) and a clear cell proportion < 25% (*p* = 0.010). SDHB negativity was also associated with tumor cell necrosis (*p* = 0.007). In PCC, SLC7A11 positivity was associated with nonnorepinephrine type (*p* = 0.008). In Kaplan-Meier analysis, patients with GLS positivity (*p* = 0.039) and SDHB negativity (*p* = 0.005) had significantly shorter overall survival in ACN. In PCC patients with a GAPP score ≥ 3, GLS positivity (*p* = 0.001) and SDHB positivity (*p* = 0.001) were associated with shorter disease-free survival, whereas GLS positivity (*p* = 0.004) was also associated with shorter overall survival.

**Conclusions:**

The expression of glutamine metabolism-related and amino acid transporter proteins in ACN and PCC is distinct and associated with prognosis.

## 1. Introduction

Typical tumors in the adrenal gland are adrenal cortical neoplasms (ACNs) arising from the adrenal cortex and pheochromocytomas (PCCs) from the adrenal medulla. ACNs comprise benign adrenal cortical adenomas (ACAs) and malignant adrenal cortical carcinomas (ACCs). ACA is a relatively common tumor, whereas ACC is an extremely rare tumor with an annual incidence of 1–2 cases per 1 million people with a highly aggressive phenotype [1, 2]. PCC is a neuroendocrine tumor that occurs in the chromaffin cells of the adrenal medulla, found in 2–8 cases per 1 million people [3]. Although ACNs and PCCs can be differentiated by disparities in the originating cells, histologic findings are similar and the behavior of these tumors cannot be adequately predicted based on histologic findings alone.

An important part of the metabolic pathway for tumor maintenance and growth is glutamine metabolism and amino acid transport. In cell culture and implanted tumor studies, cancer cells were found to metabolize glutamine better than other amino acids [4, 5]. Therefore, glutamine metabolism is considered an important metabolic phenotype of proliferating tumor cells. Glutamine metabolism contributes to two important factors in tumor cell proliferation: the production of adenosine triphosphate (ATP) and supplementation of intermediates for macromolecular synthesis. Proteins that play an important role in this glutamine metabolic pathway are amino acid transporter-2 (ASCT2), which is involved in the intracellular transport of glutamine consumed by tumor cells, glutaminase 1 (GLS1), an enzyme that converts glutamine to glutamate, and glutamate dehydrogenase (GDH), which converts glutamate into *α*-ketoglutarate to affect the TCA cycle [6–8].

Amino acids are essential for the growth and survival of tumor cells. Among them, essential amino acids such as Thr, Met, Phe, Trp, Val, Ile, Leu, and Lys must be obtained from outside the body, because they cannot be synthesized internally [9–11]. In contrast, even though nonessential amino acids can be synthesized within cells, when their demand is significantly increased by proliferating tumor cells, additional supply is required from extracellular spaces [12, 13]. Among various membrane-bound solute carrier (SLC) transporters, SLC1, SLC3, SLC6, SLC7, SLC15, SLC17, SLC18, SLC25, SLC26, SLC32, SLC36, and SLC38 are involved in the transport of amino acids. Although most amino acid transporters are expressed in a tissue- and developmentally specific manner in normal cells, the expression of specific amino acid transporters is generally higher in tumor cells, depending on the tumor type than in normal cells [9, 14–16].

Nonetheless, glutamine metabolism-related and amino acid transporter proteins in ACNs and PCCs in the adrenal gland have been poorly studied. The aim of this study was to investigate the expression of glutamine metabolism-related and amino acid transporter proteins in ACNs and PCCs and to determine the clinical implications.

## 2. Materials and Methods

### 2.1. Patient Selection

Formalin-fixed paraffin-embedded surgical tissue samples from patients diagnosed with ACN and PCC were obtained from Severance Hospital from January 2000 to December 2012. All cases were retrospectively reviewed by pathologists (Koo JS and Kim HM), and a histologic evaluation was performed on hematoxylin-eosin- (H&E-) stained slides. ACNs were examined using parameters corresponding to the Weiss criteria [17, 18], and PCCs and its catecholamine types were examined using parameters of the GAPP scoring system [19]. Disease-free survival (DFS) was calculated from the date of the first curative surgery to the date of the first locoregional or systemic relapse, or death without any relapse. Overall survival (OS) was estimated from the date of the first curative operation to the date of the last follow-up or death from any cause. Clinicopathologic parameters evaluated in each tumor included patient age at initial diagnosis, tumor recurrence, distant metastasis, and patient survival. The study was approved by the Institutional Review Board of Severance Hospital.

### 2.2. Tissue Microarray

Representative areas were selected on H&E-stained slides, and a corresponding spot was marked on the surface of the matching paraffin block. Core biopsies, 5 mm each, were taken from selected areas and placed into a 5 × 4 recipient block. More than two tissue cores were extracted from each case to minimize extraction bias. Each tissue core was assigned a unique tissue microarray location number that was linked to a database containing other clinicopathologic data.

### 2.3. Immunohistochemistry

The information of antibodies used for immunohistochemistry are listed in Supplementary Table 1. Succinate dehydrogenase subunit B (SDHB) and BCL2/adenovirus E1B 19 kDa protein-interacting protein 3 (BNIP3) staining were also performed to assess SDHB mutation and tumor hypoxia. Immunohistochemistry was performed on formalin-fixed, paraffin-embedded tissue sections using an automatic immunohistochemistry staining device (Benchmark XT, Ventana Medical System, Tucson, AZ, USA). Briefly, 5 *μ*m thick formalin-fixed paraffin-embedded tissue sections were transferred to adhesive slides and dried at 62°C for 30 minutes. Standard heat epitope retrieval was performed for 30 minutes in ethylene diamine tetraacetic acid, pH 8.0, in an autotimer. The samples were then incubated with primary antibodies. After incubation with primary antibodies, the sections were incubated with biotinylated anti-mouse immunoglobulins, peroxidase-labeled streptavidin (LSAB kit, DakoCytomation), and 3,30-diaminobenzidine. Negative control samples were processed without the primary antibody. A positive control tissue was used per the manufacturer's recommendation. Slides were counterstained with Harris hematoxylin.

### 2.4. Interpretation of Immunohistochemical Staining

All immunohistochemical markers (GLS1, GDH, SLC1A5, SLC7A5, SLC7A11, SDHB, and BNIP3) were accessed by light microscopy. Expression of the included markers was semiquantitatively evaluated using stained slides, as previously described [20]. Tumor cell staining was assessed using the following criteria: 0, negative or weak immunostaining in <1% of the tumor; 1, focal expression in 1–10% of tumors; 2, positive in 11–50% of tumors; and 3, positive in 51–100% of tumors. The entire area of each tumor was evaluated, and a score of ≥2 determined by both pathologists was defined as immunohistochemical staining positivity, whereas the score of <2 was considered negativity.

### 2.5. Statistical Analysis

Data were analyzed using IBM SPSS Statistics for Windows, Version 21.0 (Released 2012; IBM Corp., Armonk, NY, USA). To determine the statistical significance of differences, Student's *t*-test and Fisher's exact test were used for continuous and categorical variables, respectively. Statistical significance was set at *p* < 0.05. Kaplan-Meier survival curves and log-rank statistics were employed to evaluate the clinical significance of clinicopathologic markers and the DFS and OS of the patients.

## 3. Results

### 3.1. Basal Characteristics of Patients

We included a total of 115 cases of ACA and 17 cases of ACC in this study. The basal characteristics of these patients are presented in Supplementary Table 2. Patient age (*p* = 0.048) and tumor size (*p* < 0.001) were significantly different between the two groups. Parameters comprising the Weiss criteria were also significantly different between the two groups (all *p* < 0.001, Supplementary Table 2). One-hundred and eighty-nine cases of PCC were included, and the basal characteristics of these patients are presented in Supplementary Table 3.

### 3.2. Differential Expression of Glutamine Metabolism-Related and Amino Acid Transporter Proteins, SDHB, and BNIP3 in Adrenal Gland Neoplasms

**Figure 1:**
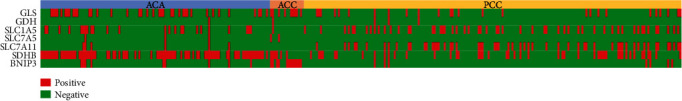
Heat map of glutamine metabolism-related and amino acid transporter proteins found in adrenal gland neoplasms (ACA: adrenal cortical adenoma; ACC: adrenal cortical carcinoma; PCC: pheochromocytoma).

**Figure 2:**
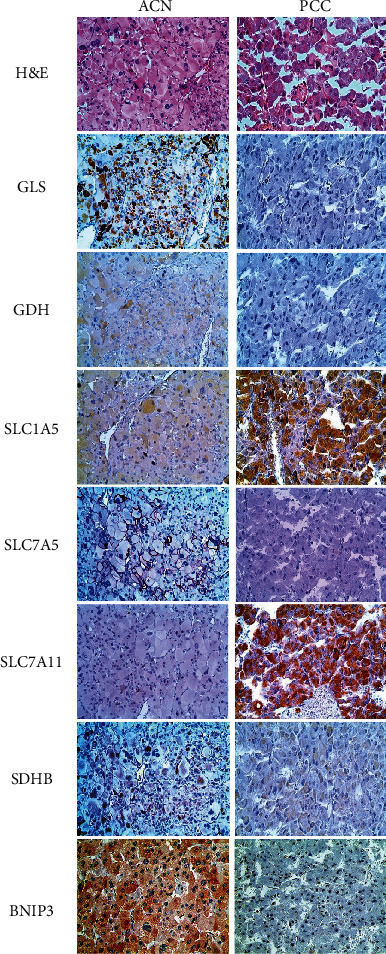
Expression of glutamine metabolism-related and amino acid transporter proteins in adrenal gland neoplasms. The expression of GLS, SLC7A5, and SDHB was higher in adrenal cortical neoplasms, whereas the expression of SLC1A5 and SLC7A11 was higher in pheochromocytomas.

GLS (*p* < 0.001), SLC7A5 (*p* = 0.007), SDHB (*p* < 0.001), and BNIP3 (*p* < 0.001) were highly expressed in ACNs, whereas SLC1A5 (*p* = 0.049) and SLC7A11 (*p* < 0.001) were highly expressed in PCCs (Table 1). The heat map regarding the expression of immunohistochemical markers and the representative images of the antibody staining are shown in Figures 1 and 2. There was no difference in the expression of glutamine metabolism-related and amino acid transporter proteins between ACAs and ACCs, and the expression of SDHB was lower and BNIP3 was higher in ACCs (Table 2).

We divided our patients with PCC into the SDHB-positive group and SDHB-negative group according to SDHB positivity. Patients with SDHB positivity revealed to have higher expression of GLS (*p* < 0.001), SLC1A5 (*p* = 0.014), and SLC7A11 (*p* = 0.001) compared to those with SDHB negativity (Table 3).

### 3.3. Correlation between the Expression Levels of Glutamine Metabolism-Related and Amino Acid Transporter Proteins and SDHB with Clinicopathologic Factors

**Figure 3:**
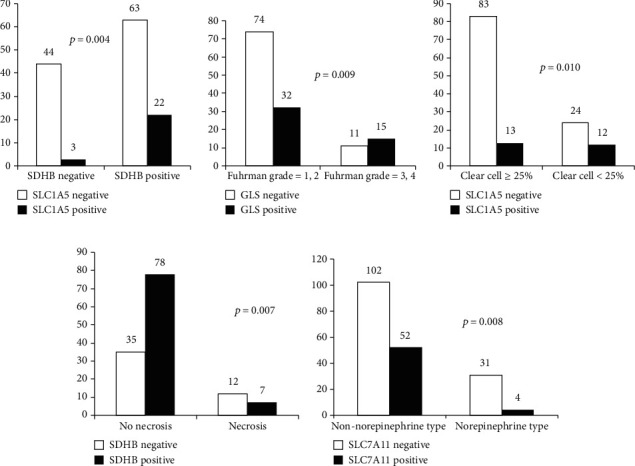
Correlation between the expression levels of glutamine metabolism-related and amino acid transporter proteins and clinicopathologic factors. In adrenal cortical neoplasms, GLS positivity was associated with a higher Fuhrman grade, and SLC1A5 positivity was associated with SDHB positivity and a clear cell proportion < 25%. Furthermore, SDHB negativity was associated with tumor cell necrosis. In pheochromocytomas, SLC7A11 positivity was associated with nonnorepinephrine types of tumors.

In ACN, GLS positivity was associated with a higher Fuhrman grade (*p* = 0.009), and SLC1A5 positivity was associated with SDHB positivity (*p* = 0.004) and a clear cell proportion of <25% (*p* = 0.010). Furthermore, SDHB negativity was associated with tumor cell necrosis (*p* = 0.007). In PCC, SLC7A11 positivity was associated with nonnorepinephrine-type tumors (*p* = 0.008) (Figure 3).

### 3.4. Expression of Glutamine Metabolism-Related Proteins and Amino Acid Transporter Proteins according to BNIP3

Considering that tumor microenvironment could be related to altered metabolism, we evaluated the expression of glutamine metabolism-related proteins and amino acid transporter proteins according to BNIP3 status. In ACN, BNIP3 positivity was associated with higher expression of GDH, SLC1A5, SLC7A5, and SLC7A11 (*p* = 0.024, *p* = 0.002, *p* < 0.001, and *p* < 0.001). Meanwhile, BNIP3 positivity was associated with the increased expression of GLS and GDH in PCC (*p* = 0.008 and *p* = 0.003) (Table 4).

### 3.5. Impact of the Expression of Glutamine Metabolism-Related and Amino Acid Transporter Proteins on Patient Prognosis in Adrenal Gland Neoplasms

**Figure 4:**
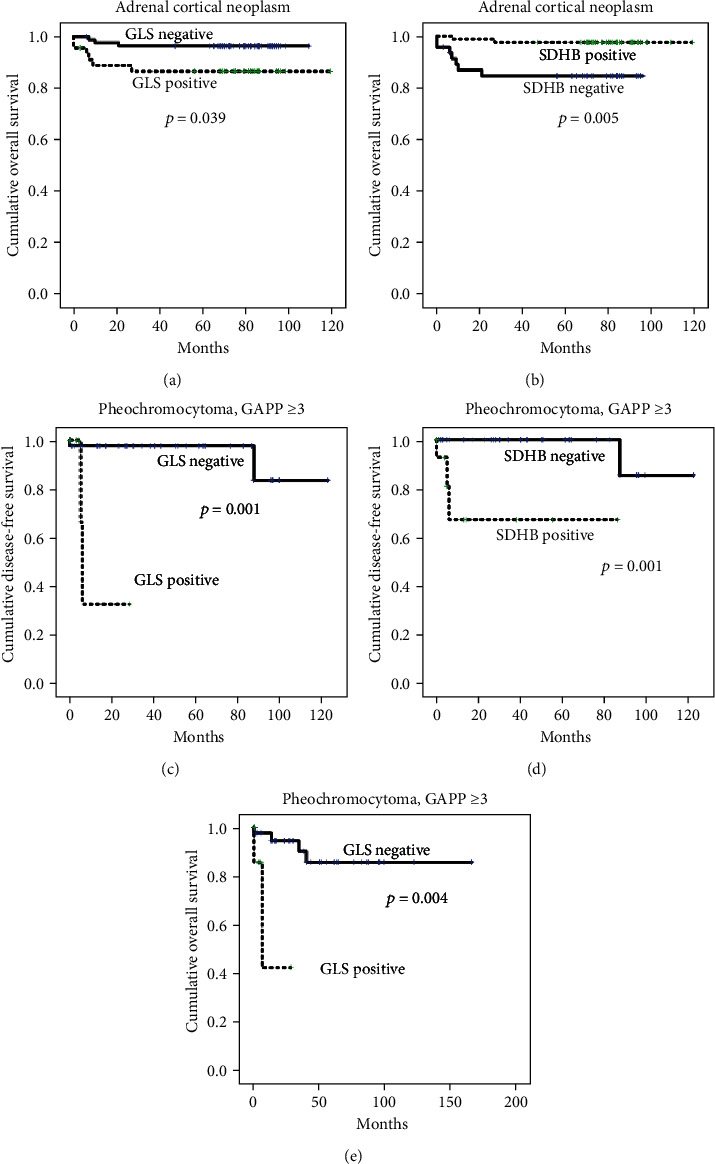
Impact of the expression of glutamine metabolism-related and amino acid transporter proteins on the prognosis of patients with adrenal gland neoplasms. In a univariate analysis, shorter overall survival was associated with GLS positivity (*p* = 0.039) and SDHB negativity (*p* = 0.005) in adrenal cortical neoplasms. In a subgroup analysis of PCC patients with GAPP scores ≥ 3, GLS positivity (*p* = 0.001) and SDHB positivity (*p* = 0.001) were associated with shorter disease-free survival in univariate analysis, whereas GLS positivity (*p* = 0.004) was associated with shorter overall survival.

In Kaplan-Meier analysis, patients with ACC had shorter DFS and OS compared to ACA (all *p* < 0.001) (Supplementary Figure 1). Additionally, GLS positivity (*p* = 0.039) and SDHB negativity (*p* = 0.005) were associated with shorter OS in ACNs. In a subgroup analysis of PCC patients with GAPP score ≥ 3, GLS positivity (*p* = 0.001) and SDHB positivity (*p* = 0.001) were associated with shorter DFS, whereas GLS positivity (*p* = 0.004) was associated with shorter OS (Figure 4).

## 4. Discussion

In the present study, we investigated the expression of glutamine metabolism-related and amino acid transporter proteins in ACNs and PCCs in the adrenal gland. Of note, GLS, SLC7A5, and SDHB were highly expressed in ACNs, and SLC1A5 and SLC7A11 were highly expressed in PCCs. Glutamine metabolism and amino acid transport in adrenal gland neoplasms have been rarely studied; therefore, it is difficult to compare our results with those of previous studies.

Several hypotheses could apply to the differential expression of glutamine metabolism-related and amino acid transporter proteins in ACNs and PCCs. Generally, ACC is detected using ^18^F-fludeoxyglucose (FDG) positron emission tomography-computed tomography (PET-CT) and PCC by ^18^F-fluorodopa (^18^F-DOPA) PET-CT [21, 22]. ^18^F-FDG PET-CT is used to detect glycolysis within a tumor, and glycolysis may be more active in ACNs than in PCCs because of the higher expression levels of GLS1 in ACNs than PCCs. Conversely, ^18^F-FDOPA is a fluorinated analog of the naturally occurring amino acid l-DOPA. It is transported into a cell by the amino acid transporter system L, which is composed of CD98hc encoded by the SLC3A2 gene and LAT1 and LAT2 encoded by the SLC7A5 and SLC7A8 genes [23]. Therefore, the expression levels of amino acid transporter proteins with similar functions, such as SLC1A5 and SLC7A11, are higher in PCCs than in ACNs.

Previous studies have shown that nearly 40% of PCCs are genetically inherited neoplasms, associated with unique metabolic abnormalities, oxygen sensing, hypermethylation, DNA repair, upregulation of specific transporters and/or receptors, and enzymes involved in the TCA cycle [24–28]. SDHx is a representative genetic variant commonly found in PCCs, and mutations in SDHx result in abnormalities in the TCA cycle that lead to altered metabolism [23, 29]. Surprisingly, we found that SDHB positivity is associated with GLS, SLC1A5, and SLC7A1 positivity in PCCs, and 75.9% of patients were found to have SDHB negativity in immunohistochemical staining. Given that SDHB gene mutation is present in approximately 6-9% of PCCs [30], this is rather contradictory to the previous results that showed negative staining for SDHB when SDHB mutation is present [31, 32]. Several possible explanations could be given regarding this inconsistency. First, to minimize interobserver bias between pathologists, we established a uniform standard to define positivity of immunohistochemical staining, which could have affected the interpretation of data. Second, SDHB staining was performed by using commercial monoclonal antibodies, and the difference of the properties of antibodies could have affected the immunohistochemical staining results. Third, a difference of ethnicity and geographic difference could be present in pheochromocytoma. However, the underlying cause of this discrepancy and its association with the expression of glutamine metabolism-related and amino acid transporter proteins should be further investigated.

Fuhrman grading is a well-known but poor prognostic indicator in adrenal gland neoplasms [17, 18]. In this study, GLS1 positivity was associated with higher Fuhrman grade and poorer prognosis in both ACNs and PCCs. Because tumor aggressiveness is associated with higher tumor metabolic activity and the expression of glutamine metabolism-related proteins, these findings imply that evaluation of GLS1 positivity could provide useful information regarding patient prognosis. However, we found that SDHB negativity, which is known to be a negative prognostic factor in PCCs [32], was associated with shorter OS in ACNs. Although the discrepant finding that was found in our study could be also related to the fact that we only included PCC with GAPP score ≥ 3 in our analysis for prognostic factors, it is also possible that the difference in defining SDHB positivity could have affected the results.

The glutamine metabolism pathway could be a possible target to control key enzymes and reduce glutamine uptake. GLS1 inhibitors such as CB-839 [33, 34], BPTES [35–37], and 968 [38–40] are currently in preclinical and clinical trials for the treatment of various cancers. Further investigations will be required to understand inhibition of GLS1 in adrenal gland neoplasms, including the effect of glutamine metabolism inhibition in ACNs, which show high glutamine metabolic activity. Alternatively, experimental evidence suggests that inhibition of amino acid transporters halts the growth of cancer cells and promotes cancer cell death [41–43]. For example, the SLC1A5 inhibitor benzylserine (Benser) hampers the proliferation of tumor cells in human gastric cancers and in melanoma cell lines [44, 45]. A selective SLC7A5 inhibitor, JPH203, has been shown to suppress cancer cell growth. In addition, JPH203 did not affect normal cells in vivo, suggesting that it can be used as a treatment agent [46, 47].

The advantage of this study is that it is the first to evaluate markers associated with glutamine metabolism in a large number of pathologically confirmed adrenal gland neoplasm, which is rare. However, this study also has several limitations. First, the expression of glutamine metabolism-related and amino acid transporter proteins was evaluated using TMA samples. In particular, as SDHB immunohistochemical staining, but not SDHB gene expression, was used to divide patients with PCC, this could have affected the interpretation of the results. Furthermore, the adoption of different cut-off to define SDHB immunohistochemical staining positivity and negativity compared to the previous studies [32, 48] should be also taken into account. Second, the clinical characteristics of neoplasms in the study were assessed retrospectively and genetic status could not be evaluated. Third, the number of ACC cases included in ACNs was small, and the heterogeneity of tumors included might have influenced the results. Finally, because tumor hypoxia is a factor related to altered glutamine metabolism, the changes in the tumor microenvironment could be also related to the expression of glutamine metabolism-related proteins and amino acid transporter proteins investigated.

Taken together, the expression of glutamine metabolism-related and amino acid transporter proteins is distinct between ACNs and PCCs and is associated with prognosis. Targeting glutamine metabolism-related and amino acid transporter proteins may hold potential therapeutic value for adrenal gland neoplasms.

## Figures and Tables

**Table 1:** Expression of glutamine metabolism-related proteins and amino acid transporter proteins in adrenal gland neoplasm.

Parameters	Total *N* = 321 (%)	Adrenal cortical neoplasm *n* = 132 (%)	Pheochromocytoma *n* = 189 (%)	*p* value
GLS				<0.001
Negative	248 (77.3)	85 (64.4)	163 (86.2)	
Positive	73 (22.7)	47 (35.6)	26 (13.8)	
GDH				0.956
Negative	316 (98.4)	130 (98.5)	186 (98.4)	
Positive	5 (1.6)	2 (1.5)	3 (1.6)	
SLC1A5				0.049
Negative	242 (75.4)	107 (81.1)	135 (71.4)	
Positive	79 (24.6)	25 (18.9)	54 (28.6)	
SLC7A5				0.007
Negative	316 (98.4)	127 (96.2)	189 (100.0)	
Positive	5 (1.6)	5 (3.8)	0 (0.0)	
SLC7A11				<0.001
Negative	259 (80.7)	126 (95.5)	133 (70.4)	
Positive	62 (19.3)	6 (4.5)	56 (29.6)	
SDHB				<0.001
Negative	189 (58.9)	47 (35.6)	142 (75.1)	
Positive	132 (41.1)	85 (64.4)	47 (24.9)	
BNIP3				<0.001
Negative	293 (91.3)	111 (84.1)	182 (96.3)	
Positive	28 (8.7)	21 (15.9)	7 (3.7)	

**Table 2:** Expression of glutamine metabolism-related proteins and amino acid transporter proteins in adrenal cortical neoplasm.

Parameters	Total *N* = 132 (%)	Adrenal cortical adenoma *n* = 115 (%)	Adrenal cortical carcinoma *n* = 17 (%)	*p* value
GLS				0.110
Negative	85 (64.4)	77 (67.0)	8 (47.1)	
Positive	47 (35.6)	38 (33.0)	9 (52.9)	
GDH				0.114
Negative	130 (98.5)	114 (99.1)	16 (94.1)	
Positive	2 (1.5)	1 (0.9)	1 (5.9)	
SLC1A5				0.141
Negative	107 (81.1)	91 (79.1)	16 (94.1)	
Positive	25 (5.9)	24 (20.9)	1 (5.9)	
SLC7A5				0.124
Negative	127 (96.2)	112 (97.4)	15 (88.2)	
Positive	5 (3.8)	3 (2.6)	2 (11.8)	
SLC7A11				0.335
Negative	126 (95.5)	109 (94.8)	17 (100.0)	
Positive	6 (4.5)	6 (5.2)	0 (0.0)	
SDHB				0.007
Negative	47 (35.6)	36 (31.3)	11 (64.7)	
Positive	85 (64.4)	79 (68.7)	6 (35.3)	
BNIP3				<0.001
Negative	111 (84.1)	106 (92.2)	5 (29.4)	
Positive	21 (15.9)	9 (7.8)	12 (70.6)	

**Table 3:** Expression of glutamine metabolism-related proteins and amino acid transporter proteins according to SDHB status in pheochromocytoma.

Parameters	SDHB negative *n* = 142 (%)	SDHB positive *n* = 47 (%)	*p* value
GLS			<0.001
Negative	130 (91.5)	33 (70.2)	
Positive	12 (8.5)	14 (29.8)	
GDH			0.153
Negative	141 (99.3)	45 (95.7)	
Positive	1 (0.7)	2 (4.3)	
SLC1A5			0.014
Negative	108 (76.1)	27 (57.4)	
Positive	34 (23.9)	20 (42.6)	
SLC7A5			n/a
Negative	142 (100.0)	47 (100.0)	
Positive	0 (0.0)	0 (0.0)	
SLC7A11			0.001
Negative	109 (76.8)	24 (51.1)	
Positive	33 (23.2)	23 (48.9)	
BNIP3			0.066
Negative	139 (97.9)	43 (91.5)	
Positive	3 (2.1)	4 (8.5)	

**Table 4:** Expression of glutamine metabolism-related proteins and amino acid transporter proteins according to BNIP3 status in adrenal gland neoplasm.

Parameters	Adrenal cortical neoplasm			Pheochromocytoma		
	BNIP3 negative *n* = 111 (%)	BNIP3 positive *n* = 21 (%)	*p* value	BNIP3 negative *n* = 182 (%)	BNIP3 positive *n* = 7 (%)	*p* value
GLS			0.080			0.008
Negative	75 (67.6)	10 (47.6)		160 (87.9)	3 (42.9)	
Positive	36 (32.4)	11 (52.4)		22 (12.1)	4 (57.1)	
GDH			0.024			0.003
Negative	111 (100.0)	19 (90.5)		181 (99.5)	5 (71.4)	
Positive	0 (0.0)	2 (9.5)		1 (0.5)	2 (28.6)	
SLC1A5			0.002			0.410
Negative	95 (85.6)	12 (57.1)		131 (72.0)	4 (57.1)	
Positive	16 (14.4)	9 (42.9)		51 (28.0)	3 (42.9)	
SLC7A5			<0.001			n/a
Negative	111 (100.0)	16 (76.2)		182 (100.0)	7 (100.0)	
Positive	0 (0.0)	5 (23.8)		0 (0.0)	0 (0.0)	
SLC7A11			<0.001			0.425
Negative	111 (100.0)	15 (71.4)		129 (70.9)	4 (57.1)	
Positive	0 (0.0)	6 (28.6)		53 (29.1)	3 (42.9)	

## Data Availability

The data used to support the findings of this study are available from the corresponding author upon request.

## Supplementary Materials

Supplementary Materials Supplementary Figure 1: impact of the expression of glutamine metabolism-related and amino acid transporter proteins on patient prognosis in adrenal gland neoplasm. In Kaplan-Meier analysis, patients with ACC had shorter DFS and OS compared to ACA (all *p* < 0.001). Supplementary Table 1: source, clone, and dilution of used antibodies. Supplementary Table 2: basal characteristics of adrenal cortical neoplasm. Supplementary Table 3: basal characteristics of pheochromocytoma.
