# Supplementary material for: Expression of Glutamine Metabolism-Related and Amino Acid Transporter Proteins in Adrenal Cortical Neoplasms and Pheochromocytomas
Source: Dis Markers. 2021 Jan 5;2021:8850990. doi: 10.1155/2021/8850990 (PMC7806379; doi:10.1155/2021/8850990)
Supplement: Supplementary Materials — Supplementary Figure 1: impact of the expression of glutamine metabolism-related and amino acid transporter proteins on patient prognosis in adrenal gland neoplasm. In Kaplan-Meier analysis, patients with ACC had shorter DFS and OS compared to ACA (all p < 0.001). Supplementary Table 1: source, clone, and dilution of used antibodies. Supplementary Table 2: basal characteristics of adrenal cortical neoplasm. Supplementary Table 3: basal characteristics of pheochromocytoma. [file 8850990.f1.docx]

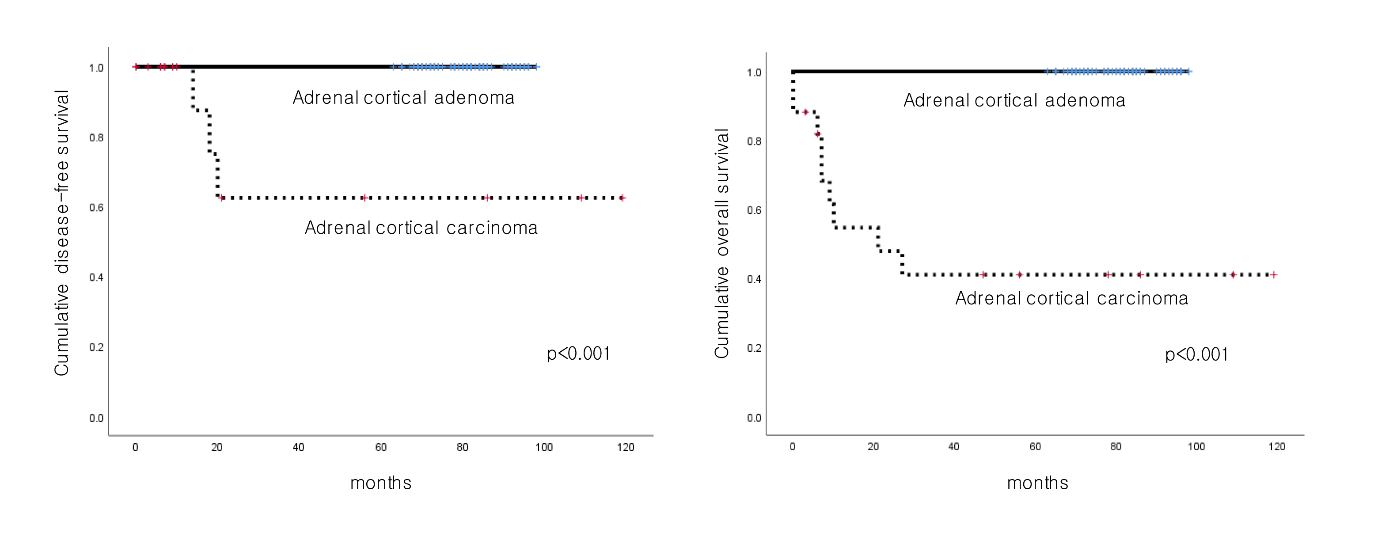
**Supplementary figure 1.** Impact of the expression of glutamine metabolism-related and amino acid transporter proteins on patient prognosis in adrenal gland neoplasm. In Kaplan-Meier analysis, patients with ACC had shorter DFS and OS compared to ACA (all p < 0.001).

| **Supplementary table 1.** Source, clone, and dilution of used antibodies | | | |
| --- | --- | --- | --- |
| Antibody | Clone | Dilution | Company |
| GLS1 | polyclonal | 1:50 | Abcam, Cambridge, UK |
| GDH | polyclonal | 1:100 | Abcam, Cambridge, UK |
| SLC1A5 | polyclonal | 1:100 | Abcam, Cambridge, UK |
| SLC7A5 | monoclonal (EPR17573) | 1:500 | Abcam, Cambridge, UK |
| SLC7A11 | polyclonal | 1:200 | Abcam, Cambridge, UK |
| SDHB | monoclonal (21A11AE7) | 1:200 | Abcam, Cambridge, UK |
| BNIP3 | monoclonal (ANa40) | 1:200 | Abcam, Cambridge, UK |
|  | | | |

| **Supplementary Table 2**. Basal characteristics of adrenal cortical neoplasm | | | | |
| --- | --- | --- | --- | --- |
| Parameters | Total  N=132 (%) | Adrenal cortical adenoma n=115 (%) | Adrenal cortical carcinoma n=17 (%) | p-value |
| Age  (year, mean±SD) | 47.5±145 | 48.4±12.2 | 41.0±25.1 | **0.048** |
| Sex |  |  |  | 0.107 |
| Male | 40 (30.3) | 32 (27.8) | 8 (47.1) |  |
| Female | 92 (69.7) | 83 (72.2) | 9 (52.9) |  |
| Tumor size  (cm, mean±SD) | 3.6±3.7 | 2.5±1.3 | 10.9±5.8 | **<0.001** |
| Fuhrman grade |  |  |  | **<0.001** |
| 1, 2 | 106 (80.3) | 102 (88.7) | 4 (23.5) |  |
| 3, 4 | 26 (19.7) | 13 (11.3) | 13 (76.5) |  |
| Mitosis |  |  |  | **<0.001** |
| ≤5/50HFPs | 122 (92.4) | 115 (100.0) | 7 (41.2) |  |
| >5/50HFPs | 10 (7.6) | 0 (0.0) | 10 (58.8) |  |
| Atypical mitosis |  |  |  | **<0.001** |
| Absent | 121 (91.7) | 114 (99.1) | 7 (41.2) |  |
| Present | 11 (8.3) | 1 (0.9) | 10 (58.8) |  |
| Clear cell proportion |  |  |  | **<0.001** |
| ≥25% | 96 (72.7) | 95 (82.6) | 1 (5.9) |  |
| <25% | 36 (27.3) | 20 (17.4) | 16 (94.1) |  |
| Diffuse architecture |  |  |  | **<0.001** |
| Absent | 117 (88.6) | 111 (96.5) | 6 (35.3) |  |
| Present | 15 (11.4) | 4 (3.5) | 11 (64.7) |  |
| Necrosis |  |  |  | **<0.001** |
| Absent | 113 (85.6) | 113 (98.3) | 0 (0.0) |  |
| Present | 19 (14.4) | 2 (1.7) | 17 (100.0) |  |
| Venous invasion |  |  |  | **<0.001** |
| Absent | 126 (95.5) | 115 (100.0) | 11 (64.7) |  |
| Present | 6 (4.5) | 0 (0.0) | 6 (35.3) |  |
| Sinusoidal invasion |  |  |  | **<0.001** |
| Absent | 126 (95.5) | 115 (100.0) | 11 (64.7) |  |
| Present | 6 (4.5) | 0 (0.0) | 6 (35.3) |  |
| Capsular invasion |  |  |  | **<0.001** |
| Absent | 117 (88.6) | 111 (96.5) | 6 (35.3) |  |
| Present | 15 (11.4) | 4 (3.5) | 11 (64.7) |  |
| Weiss total score |  |  |  | **<0.001** |
| < 4 | 117 (88.6) | 115 (100.0) | 2 (11.8)* |  |
| ≥ 4 | 15 (11.4) | 0 (0.0) | 15 (88.2) |  |
| Recurrence | 3 (2.3) | 0 (0.0) | 3 (17.6) | **<0.001** |
| Distant metastasis | 7 (5.3) | 0 (0.0) | 7 (41.2) | **<0.001** |
| Patient death | 9 (6.8) | 0 (0.0) | 9 (52.9) | **<0.001** |

| **Supplementary Table 3**. Basal characteristics of pheochromocytoma | |
| --- | --- |
| Parameters | Total, N=189 (%) |
| Age (year, mean±SD) | 48.1±13.7 |
| Sex |  |
| Male | 73 (38.6) |
| Female | 116 (61.4) |
| Tumor size (cm, mean±SD) | 5.0±3.4 |
| Histologic pattern |  |
| Zellballen | 165 (87.3) |
| Non-Zellballen | 24 (12.7) |
| Cellularity |  |
| Low | 11 (5.8) |
| Moderate | 162 (85.7) |
| High | 16 (8.5) |
| Comedo necrosis |  |
| Absent | 189 (100.0) |
| Present | 0 (0.0) |
| Vascular or capsular invasion |  |
| Absent | 129 (68.3) |
| Present | 60 (31.7) |
| Ki-67 labeling index (%) |  |
| <1 | 139 (73.5) |
| 1-3 | 38 (20.1) |
| >3 | 12 (6.3) |
| Catecholamine type |  |
| Non-norepinephrine type | 154 (81.5) |
| Norepinephrine type | 35 (18.5) |
| GAPP score |  |
| 0-2 (well-differentiated type) | 138 (73.0) |
| 3-6 (moderately differentiated type) | 50 (26.5) |
| 7-10 (poorly differentiated type) | 1 (0.5) |
| Tumor recurrence | 5 (2.6) |
| Distant metastasis | 7 (3.7) |
| Patient death | 11 (5.8) |
